# Advanced Preparation Makes Research in Emergencies and Isolation Care Possible: The Case of Novel Coronavirus Disease (COVID-19)

**DOI:** 10.4269/ajtmh.20-0205

**Published:** 2020-03-30

**Authors:** David M. Brett-Major, Elizabeth R. Schnaubelt, Hannah M. Creager, Abigail Lowe, Theodore J. Cieslak, Jacob M. Dahlke, Daniel W. Johnson, Paul D. Fey, Keith F. Hansen, Angela L. Hewlett, Bruce G. Gordon, Andre C. Kalil, Ali S. Khan, Mark G. Kortepeter, Christopher J. Kratochvil, LuAnn Larson, Deborah A. Levy, James Linder, Sharon J. Medcalf, Mark E. Rupp, Michelle M. Schwedhelm, James Sullivan, Angela M. Vasa, Michael C. Wadman, Rachel E. Lookadoo, John-Martin J. Lowe, James V. Lawler, M. Jana Broadhurst

**Affiliations:** 1 University of Nebraska Medical Center/Nebraska Medicine, Omaha, Nebraska;; 2 United States Air Force School of Aerospace Medicine, Dayton, Ohio

## Abstract

The optimal time to initiate research on emergencies is before they occur. However, timely initiation of high-quality research may launch during an emergency under the right conditions. These include an appropriate context, clarity in scientific aims, preexisting resources, strong operational and research structures that are facile, and good governance. Here, Nebraskan rapid research efforts early during the 2020 coronavirus disease pandemic, while participating in the first use of U.S. federal quarantine in 50 years, are described from these aspects, as the global experience with this severe emerging infection grew apace. The experience has lessons in purpose, structure, function, and performance of research in any emergency, when facing any threat.

The University of Nebraska Medical Center and its clinical partner Nebraska Medicine (UNMC/NM) were confronted with a unique set of circumstances at the start of the U.S. experience with novel coronavirus disease (COVID-19) that highlighted core lessons regarding research in emergencies that might be applied in any location, and to any disease. Ultimately, UNMC/NM conducted a prospective, observational cohort study beginning with COVID-19–infected persons in isolation care. The rapidly traveled road to this study had many curves.

The University of Nebraska Medical Center and its clinical partner Nebraska Medicine are accustomed to responding to public health emergencies. It cared for patients with Ebola virus disease from West Africa; received persons exposed to other high consequence pathogens; established and maintained the Nebraska Biocontainment Unit; with partners Emory University, Bellevue Hospital, and the CDC, led the National Ebola Training and Education Center (NETEC); launched the National Quarantine Unit funded by the Health and Human Services (HHS) Assistant Secretary of Preparedness and Response office; and established the Global Center for Health Security to coordinate its other national and international health emergency initiatives. The biocontainment unit was established in the aftermath of outbreaks of severe acute respiratory syndrome coronavirus (SARS-CoV) and avian influenza A in the early 2000s, getting its first use in the 2014–2016 West African Ebola virus disease epidemic. The unit has critical care capabilities. The quarantine unit has airborne precaution capabilities but was designed to accommodate groups of individuals who are not ill, a need suggested by returned healthcare workers following occupational exposures in the West African epidemic. Even for an institution with experience in both timely research and management of patients with highly communicable diseases, the conditions under which coronavirus disease (COVID-19) was introduced to the United States and the pervasive challenges of patient-centered research in emergencies complicated considerations (Figure 1).

**Figure 1.**
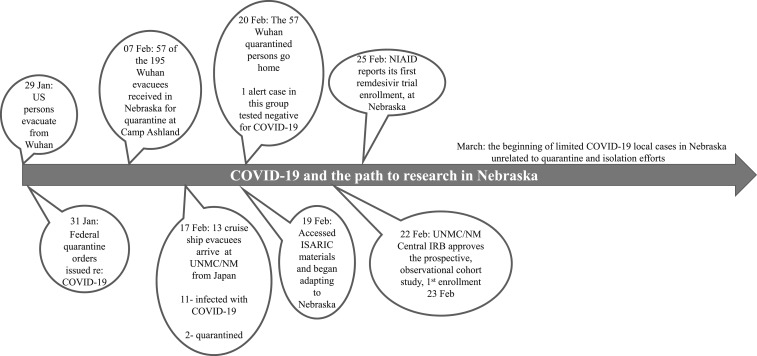
Time line of the path to coronavirus disease research at the University of Nebraska Medical Center and its clinical partner Nebraska Medicine.

## QUARANTINE IS A VERY DIFFERENT RESEARCH SETTING THAN ISOLATION CARE

Nebraska’s first guests related to COVID-19 were 57 men, women, and children evacuated from Wuhan, China, and in quarantine. They arrived on federally chartered aircraft and passed through carefully arranged screening checks manned by CDC officials. Then, uniformed public health service personnel from other agencies within HHS and managed by the Assistant Secretary for Preparedness and Response office provided support. Security was present, including U.S. marshals. Everyone—including those who had placed a large Nebraska welcome banner at the airport, deposited gift baskets in dormitory rooms, or wielded thermometers—wanted these persons to arrive well, stay well, and feel welcome. These individuals were glad to be back in the United States. They did not want others to become infected with COVID-19 if, in fact, they proved to be ill. Nonetheless, they were constrained by schedule, location, and physical and human barriers until their departure from quarantine. On the verge of the departure of the evacuees from China, UNMC/NM received a mix of 11 isolated (infected) and two quarantined (not known to be infected) individuals evacuated by federal authorities from a cruise ship in Japan.

The evacuees from Wuhan were among the first in the United States to be placed under quarantine by the federal government under new authorities established in the 2017 revision of the Code of Federal Regulations^1,2^ (Box 1). Federal quarantine orders—the first such use in over 50 years—presented contextual challenges. In general, U.S. quarantine stations exist at major points of entry, such as at large international airports, where small numbers of sentinel cases of an emerging disease are thought to be most likely to be first encountered in the United States.^3^ In late 2019, UNMC/NM opened the first national quarantine unit. This twenty-bed unit is designed to host larger numbers of quarantined persons than existing, smaller quarantine stations. It is colocated with a national training resource for public health emergency personnel, in close proximity to the Nebraska Biocontainment Unit, to enable more advanced care if needed.^4^ This large group of 57 persons, however, were managed by federal authorities at Camp Ashland—a Nebraska Army National Guard base outside of Omaha—with some logistics support from UNMC/NM.

**Box 1:** A revised quarantine law’s first use


On January 31, 2020, the CDC issued a federal mandatory quarantine order for 195 Americans evacuated out of Wuhan, China, on January 29.^18^ Effective February 2, Health and Human Services declared a mandatory quarantine for any U.S. citizens or permanent residents returning to the United States who had returned from the Hubei Province of China in the previous 14 days. In addition, all U.S. citizens and permanent residents returning from mainland China were required to undergo two weeks of self-monitoring.
This marked the first time in over 50 years that mandatory federal quarantine had been invoked under the CDC’s jurisdiction. By contrast, federal isolation orders have been comparatively common. Isolation differs from quarantine in that isolation requires infection with a quarantinable, communicable disease, not just exposure. Between 2005 and 2016, the CDC issued 12 federal isolation orders,^19^ relying mainly on port-of-entry screening.
Historically, state and local health departments have executed most quarantine orders. In 2019, to decrease the spread of measles in California, Los Angeles County did so for more than 200 individuals at two college campuses.^20^ The quarantine measure met little resistance. Other state orders have not been so well received. In 2014, New Jersey issued a quarantine order for a nurse returning to the United States after caring for Ebola patients in Sierra Leone. Afterward, the nurse filed suit in federal court, stating that New Jersey had violated her constitutional rights to liberty and due process. She later dropped her suit, settling in favor of changes to the state’s quarantine regulations, including provisions for the right to counsel, notice of hearings, visitor rights, and the right to privacy.^21^ The case suggests where some points of friction may arise as the CDC continues its COVID-19 orders.
Coronavirus Disease-19 quarantines also were the first to test recently updated regulations. In 1967, quarantine authority shifted to the CDC for cases involving ports of entry, with interstate quarantine added to the CDC’s jurisdiction in 2000. Related regulations have had several updates, most recently in 2017, with a stated focus on individuals’ due process rights.
Some anticipated issues include mandatory reassessment of quarantine cases, social distancing practices, compensation for lost wages, and payment for the care and treatment of quarantined individuals. Under the current regulations, any federal isolation or quarantine order must be reassessed within 72 hours of issuance of the order, seemingly impractical in light of large numbers of related cases, if conducted individually. It may be impossible to house each person alone, despite the consequences for housemates if the person is infected. In addition, regulations do not expressly direct payment for the care and treatment of individuals subject to a federal quarantine. These costs may include diagnostic testing. The regulations allow that the director of the CDC may authorize payment for such care and treatment, but that payment is in the CDC’s sole discretion. This language leaves matters of payment open to interpretation and negotiation, which may be a hindrance to real-time decision-making.
Many of these issues relate to differences between small-scale quarantines and the additional challenge of larger scale events, as relevant for COVID-19. As this health emergency evolves, ambiguous guidelines, combined with the unprecedented nature and scale of this quarantine, could impact the operational response. The CDC is in a unique situation to take precedent-setting action, establishing new standards for how federal quarantine should occur in the United States for many years to come.

By the time that the quarantined persons from Wuhan arrived, experts had already considered the possibility that SARS-CoV-2 might shed before symptoms, facilitating its ability to achieve sustained human-to-human transmission.^5^ For this reason, UNMC/NM initially sought to test asymptomatic individuals to inform their case management and how they were housed. However, a consensus regarding the advisability of testing could not be reached with authorities because of concerns regarding their personal autonomy (whether the quarantined persons understood the implications of testing and could make a choice freely) and uncertainty about what to do about isolated negative test results. Testing was not pursued. In the end, none of the quarantined evacuees from Wuhan demonstrated clinical evidence of COVID-19.^6^ The question of scope of presymptomatic shedding remained unanswered.

Toward the end of that quarantine, on 17 February, UNMC/NM received a group from a cruise ship in Japan comprised mostly of COVID-19–infected persons.^7^ The infected individuals were under federal isolation orders as opposed to quarantine; they were known to be infected. Whether simply being observed in the setting of few or no symptoms, or more ill and in need of hospital level care, they were housed at UNMC/NM. By that time, Asia had accumulated many cases, and a literature base was developing.^8,9^ Nonetheless, cases in the United States remained few, and availability of information and specimens from affected areas in Asia that were relevant to medical countermeasure development was limited. This prompted UNMC/NM to launch its own research initiative for the prospective assessment of patients. It did so against a backdrop of initial hesitancy because of complex issues of patient autonomy under federal orders, interagency jurisdiction challenges as different governmental actors exercised their perceived obligations for oversight, and known larger patient populations in other countries that might make local research less important.

## GOOD SCIENCE IN EMERGENCIES HELPS RISK MANAGEMENT DECISION-MAKING

Once the decision to initiate research was made, one of the immediate questions was on what? UNMC/NM participated in the National Institute of Allergy and Infectious Diseases (NIAID) studies of drug therapy against Ebola virus disease, and this collaboration continued in support of an adaptive randomized controlled trial with the antiviral drug remdesivir.^10,11^ As the first institution to initiate this trial for COVID-19 patients in the United States, UNMC/NM assisted expansion of the trial to additional sites via its rapid response central Institutional Review Board (IRB) mechanism for the NETEC Special Pathogens Research Network.

Finding an effective drug, however, is not the only purpose of doing research in emergencies. Early in response efforts, a critical questions and ethics committee was formed. Pulling from a multidisciplinary base, its purpose was providing a space for leadership and others to air questions, concerns, and challenges that might represent an obstruction to effective risk management—a space to reflect amidst an otherwise operationally fast-paced environment. Fielded questions were sometimes narrow and sometimes broad. They often highlighted uncertainty about the disease itself, which limited the ability to make evidence-based decisions. This process facilitated stakeholders coming together to start pursuing answers (Box 2). The committee also undertook a survey of research associated with the response and started to link risk management challenges with sources of information that might assist decision-making. Several needs were evident as research planning discussions ensued (Box 3). Importantly, these discussions led to a broad picture of how a platform for research might be applied, and a prospective, observational cohort study design was selected.

**Box 2:** Critical Questions and Ethics Vignette


The University of Nebraska Medical Center and its clinical partner Nebraska Medicine established a Critical Questions and Ethics committee immediately before experiencing its first COVID-19 patients. This allowed decision-makers and implementers alike a space in which to air concerns based on unanswered questions or perceived operational or organizational risks. The committee was advisory in nature. One such question asked how best to prioritize N95 respirators that were anticipated to be in short supply. The conversation revolved around fit-testing requirements. At a center like UNMC/NM, several hundred respirators are consumed each year in quantitative fit testing for staff who have newly arrived, or for required periodic testing. Logistical, ethical, legal, and operational considerations included finding the right balance between the need for appropriate fit—especially if at high risk of SARS-CoV-2 exposure, differences in regulatory intent for fit testing and a more rigorous standard applied by the university, and preconceived notions of need, practice, and requirements. Several small program adjustments were thought to have promise. These were reevaluating nondestructive or qualitative fit testing, using a survey to enable a longer interval before retesting, and prioritizing new employees and areas with higher risk for encounters with ill patients. Important research avenues emerged, and this process highlighted the need for interdisciplinary approaches. Environmental hygiene, logistics, and implementation science aims all arose from the conversation in ways that might not otherwise have emerged. Decision-related knowledge needs relevant to the prospective, observational cohort study described in this article have included viral shedding dynamics, clinical course relevant to resource demand, and the horizon of available medical countermeasures and their development.

**Box 3:** Important features of a research cohort study during any health emergency

• Risk identification and characterization of the disease in patients • Hypotheses generation with a potential to impact patient- and community-centered outcomes • Continual patient population assessment so that work to test hypotheses is best designed and fundamental processes are well framed and practiced • Flexibility to interact with clinical care and public health teams when the study could provide meaningful information, particularly in real time or near real time, including coordination with environmental sampling and testing • Potential to explore data, specimens, and the results of analysis over time, to include the potential for cooperative work with partners across stakeholder groups • Flexibility to adjust the schedule of events when exigencies such as when infection prevention and control posture or immediate patient interests require changes • Durable rather than fleeting investment of time and other resources, so that all are ready when new health emergencies present

## HAVING PREEXISTING PROTOCOLS IS VERY HELPFUL

Fortunately, the International Severe Acute Respiratory and Emerging Infection Consortium (ISARIC) and the World Health Organization (WHO) had been working on a protocol for just such a prospective, observational cohort study for several years.^12^ Known as the Clinical Characterization Protocol for Severe Emerging Infections, it represents a longitudinal effort to generate and keep updated an internationally harmonized protocol for the evaluation of emerging infections.^13^

The existence of a well-developed protocol with case report form, informed consent documents, and other supporting materiel had immediate advantages. From a science management perspective, the most striking aspect was that the well-documented evolution of the protocol simplified local scientific review requirements. Moreover, it was easier to edit than to initiate writing. UNMC/NM changes to documents reflected technical preferences, differences in local law or institutional requirements, or using the documents in a referral academic center rather than a resource-limited setting. Overall, the ISARIC materials saved at least several days in the process and provided important guideposts.

## STRONG STRUCTURES MUST ALSO BE FACILE

UNMC/NM have several unique features in its IRB. The IRB has technical breadth, a dedicated pool of community representatives, and a process for rapid review. In addition, the university has invested in this office so that when called upon for rapid reviews, there are sufficient highly committed staff to participate in management and oversight of the process, as well as consultation with petitioning investigators. Just as importantly, the IRB has experience with reviews in emergencies and related exercises. It also has worked through how to facilitate cooperative research through its central IRB mechanism for the Special Pathogens Research Network, comprising 10 academic centers that serve as regional referral isolation care hospitals.^14^ The regulatory process reflects a general posture toward discovery in parallel with clinical care shared across its network partners.^15^ Operational efficiency such as that provided by the central IRB was impactful in ensuring the window of opportunity was not lost.

A curious, structural aspect of research preparedness that became clear while assisting other sites considering adoption of the UNMC/NM prospective, observational cohort study was the importance of routine access. UNMC/NM and other referral location personnel regularly access isolation care spaces in training and response activities, as well as participate in community coordination in the management of patients who may have an infection with a high consequence pathogen. Consequently, the primary pool of investigators needed at the bedside and in the laboratory are readily able to undertake practices and follow procedures within containment areas, including Institutional Biosafety Committee–appropriate laboratory spaces. Consequently, when an emergency such as the COVID-19 pandemic occurs, the work is feasible.

In each emergency, some structures preexist, some must be applied anew, and priorities must be set.^16^ For COVID-19 with a remdesivir drug trial from NIAID on site and its potential to impact care generally, UNMC/NM tiered offers of enrollment to its patients, first screening for the drug trial before considering other research on a given patient.

## EVERYONE HAS REQUESTS THAT MUST BE MANAGED

In just over a week from conception, in the beginnings of delivering isolation care, seven participants with COVID-19 infections were enrolled in a prospective, observational cohort study for severe emerging infections. The study rapidly accumulated both prospectively collected and residual clinical specimens. In contrast to accumulated experiences in Asia and some other affected areas, the cohort was small. Nonetheless, it captured high-quality specimens coupled with data of value to researchers and product developers in the midst of a new emerging infectious disease.

The UNMC/NM prospective observational cohort study incorporated a tissue bank, allowing the later use of study specimens. In accordance with regulatory requirements, it has a governance structure. A Priorities Steering Committee was established immediately, including some members of the investigator group and other stakeholders. A formal request process for use of data and specimens was instituted, and a request tracker quickly filled with governmental, academic, and industry requests that were as varied as they were rapid. The committee adopted a long view for use of the tissue bank, recognizing the need to balance exigent with future possibilities for use. It recognized the importance of transparency, cooperative work meeting aims not achievable by smaller groups or individuals, and the need to facilitate meaningful innovation that might not be served by other initiatives.

In emergencies, however, long views may not be popular, requests are not always rational, disclosures and realistic assessments are not often available on the actual utility of an experiment in the context of the emergency at hand, and respect for autonomy and appreciation of the intent of a gift of data and sample by a patient are not always appreciated. However, that investigators believe in their work and seek to advance discovery is important for both patients and science. Being arbiters of limited resources in this context means that everyone must make compromises.

## FUNDING IS COMPLICATED

UNMC/NM launched the prospective, observational cohort study without external funding. Cohort studies often are challenging to fund. Research dollars tend not to align with durable, multi-threat capabilities.^17^ Such studies may be supported in part through sub-study funding, for instance, to test a particular device and assay in the laboratory on samples from cohort members. Without new funding solutions, the development of valuable cohorts may not be possible.

## SUMMARY

The University of Nebraska Medical Center and its clinical partner Nebraska Medicine quickly established a prospective, observational cohort study for severe emerging infections during the 2020 COVID-19 emergency, while supporting national quarantine and isolation care activities and launching an NIAID randomized, controlled drug trial. This was possible thanks to preexisting resources from the international community and durable partners, as well as structures that support research review and execution with intrinsic aspects that allow flexibility. Studies in emergencies must be designed in ways mindful of the context in which they start, and yet have a long view. As in all science, aims must be clear, mechanisms for governance present, and opportunities for reflection and input encouraged. Despite challenges and sometimes a lack of external funding support, research is a worthwhile undertaking to advance understanding and seek risk management solutions.
